# Prolonged mask wearing changed nasal microbial characterization of young adults during the COVID-19 pandemic in Shanghai, China

**DOI:** 10.3389/fimmu.2023.1266941

**Published:** 2023-10-16

**Authors:** Guoxiu Xiang, Kai Xu, Ying Jian, Lei He, Zhen Shen, Min Li, Qian Liu

**Affiliations:** ^1^ Department of Laboratory Medicine, Renji Hospital, School of Medicine, Shanghai Jiao Tong University, Shanghai, China; ^2^ Faculty of Medical Laboratory Science, College of Health Science and Technology, School of Medicine, Shanghai Jiao Tong University, Shanghai, China

**Keywords:** masks, nasal microbiota, 16S rRNA gene sequencing, COVID-19, young adults

## Abstract

**Background:**

Face masks have become a common sight during the Coronavirus Disease 2019 (COVID-19) pandemic in many countries. However, the impact of prolonged face mask wearing on nasal microbiota of healthy people is not fully understood.

**Methods:**

In this study, we compared the nasal microbiota of 82 young adults who wore face masks for an extended period of time to 172 mask-free peers from the same school recruited before the COVID-19 pandemic via 16S ribosomal RNA gene sequencing. Diversity, composition, and function of nasal microbiota between the two groups were analyzed. Prevalence of commensal bacteria colonized in the nasal cavity was determined by culture-based analysis.

**Results:**

We observed that prolonged face mask wearers had significantly different nasal microbial characterization and metabolic function compared to mask-free controls from 2018. Specifically, the nasal microbiota of the prolonged mask wearers displayed increased abundance of *Staphylococcus*, *Pseudoalteromonas*, *Corynebacterium*, etc. Meanwhile, the abundance of several genera including *Bacteroides*, *Faecalibacterium*, and *Agathobacter* was decreased. Moreover, we observed that COVID-19 infection history did not affect the composition of nasal microbiota significantly. Additionally, the culture-based analysis revealed that *Staphylococcus aureus* and *Corynebacterium accolens* increased, and *Staphylococcus epidermidis* decreased in the nasal cavity of prolonged mask wearers.

**Conclusions:**

Overall, our study suggests that prolonged face mask wearing can significantly alter the nasal microbiota.

## Introduction

The nasal microbiota plays a crucial role in the maintenance of respiratory health, and disturbance in its composition and structure may lead to various respiratory disorders (1). It has been reported that chronic rhinosinusitis and chronic neurological diseases are linked to dysbiosis of nasal microbiota (2–4). Opportunistic pathogens such as *Staphylococcus aureus* or *Moraxella catarrhalis* often colonize in the nasal cavity, and lead to respiratory or systemic infections (5), while the commensal bacteria from nasal microbiota, including *Staphylococcus*, *Dolosigranulum*, *Corynebacterium*, and other bacterial genera, have a positive impact on respiratory health and immune function (6).

The Coronavirus Disease 2019 (COVID-19) pandemic has brought significant changes in our daily life, and wearing masks has become a universal practice in many countries. While masks are an effective way to prevent the transmission of droplets, they can also have potential adverse effects on respiratory physiology, such as facial acne, halitosis, and headache (7). Masks also create a warm and humid microenvironment, which can lead to microbiome dysbiosis and related medical problems (8). Studies have shown that prolonged mask wearing increases the incidence of facial skin problems and reduces the diversity of facial microbiota (9, 10). Additionally, long-term mask wearing may increase halitosis levels due to the presence of salivary microorganisms such as *Porphyromonas gingivalis* and *Treponema denticola* (11). However, there is still limited information on whether extended mask wearing alters the diversity, composition, and structure of the nasal microbiota.

Understanding the potential impact of mask wearing on the nasal microbiota is important for maintaining respiratory health and preventing any adverse effects. In the study, we compared the diversity, composition, and function differences of nasal microbiota between extended mask wearing healthy young adults and mask-free peers from the same school recruited before the COVID-19 pandemic.

## Materials and methods

### Recruitment of human subjects

This study was approved by the ethics committee of Renji Hospital, School of Medicine, Shanghai Jiaotong University, Shanghai, China (protocol 2017001). All individuals provided informed consent. Healthy young adults aged between 18 and 25 years old living in the same school in Shanghai, China were recruited in January 2018 and February 2023, respectively. General information including age, sex, medical history, and mask wearing status of the previous 6 months was collected by questionnaires. Each participant in 2023 has claimed to be a long-term mask user and has been wearing masks since January 2020, the early stages of the COVID-19 pandemic. Young adults recruited in 2018, before the COVID-19 pandemic, were regarded as mask-free controls. A total of 82 prolonged mask wearers and 172 mask-free controls fulfilling the following criteria were enrolled: (1) no evidence of opportunistic respiratory tract infections or respiratory diseases; (2) no history of antibiotic intake within the previous 30 days; (3) no history of diabetes, malnutrition, immunodeficiencies, or cancers; and (4) no history of smoking. Specimens were collected by professionals using a sterilized cotton swab (COPAN LQ Stuart Transport Swab; COPAN Italia). The swabs were submerged into 1 mL of sterile saline preservation solution immediately for further use. More details on the sampling process are provided in **Data Sheet 1**.

### Culture-based analysis

Nasal swabs were fully vortexed in 1 mL of sterile saline for 2 min. Samples (100 μL) from each swab were plated on 5% sheep blood agar and incubated at 37°C for 24 h. Eight random colonies were isolated and identified by MALDI-TOF-MS (Bruker Daltonics, Bremen, Germany) in each sample. All identified bacterial isolates are listed in **Data Sheet 2**.

### DNA extraction and 16S rRNA gene sequencing

DNA was extracted as follows. Nasal swabs were fully vortexed in 1 mL of sterile saline for 2 min. Bacteria pellet was collected by centrifugation for 10 min at 12,000 rpm. The pellets were resuspended in 180 μL of Buffer ATL (QIAamp DNA Mini Kit, QIAGEN 51306) with 5 μL of lysozyme (50 mg/mL, Sigma L6876) and 5 μL of lysostaphin (1 mg/mL, Sigma L4402) for 30-min incubation at 37°C. Then, DNA was extracted according to the manufacturer’s instructions. Hypervariable V3 and V4 regions of the bacterial 16S rRNA gene were amplified using the primer pair 341F (5′-CCTACGGGNBGCASCAG-3′) and 805R (5′-GACTACHVGGGTATCTAATCC-3′). The amplicons were then sequenced on the Illumina platform with 30,000 reads volume by the same sequencing company.

### Amplicon sequence analysis

The data derived from sequencing were processed using QIIME for further analyses (12). Only reads with high-quality bases (Phred quality score≥25) exceeding 90% were retained for analysis. Chimeras were detected and filtrated by USEARCH 8.0 implemented in QIIME. Filtrated sequences were clustered into operational taxonomic units (OTUs) at a similarity level of 99% using the Uclust algorithm implemented in QIIME. A total of 986 OTUs were obtained and the average number of sequences per sample was 24,708. Taxonomic annotation was performed using the SILVA v138.1 database (13). Low-abundance OTUs fewer than 2 counts in at least 10% of samples were filtered. Taxonomic α-diversity was estimated using Shannon and Simpson indices, and compared using the Mann–Whitney *U* test. β-diversity between groups was measured using principal coordinate analysis (PCoA) based on Bray–Curtis dissimilarity and compared using the permutational multivariate analysis of variance (PERMANOVA) method.

### Differential analysis of taxa and metadata

Microbiome taxonomic abundance data were analyzed using the Mann–Whitney *U* test or the Kruskal–Wallis *H* test to identify differentially abundant features. MaAsLin2 was used to adjust covariates including age and sex (14). KEGG pathway abundance was predicted using PICRUSt2 (15). The normalized abundance value of the predicted pathways for each sample was visualized in a heatmap.

### Statistical analyses

All statistical analyses were performed in R. Unpaired, two-tailed *t*-tests were used for the comparison of continuous variables. Chi-square tests or Fisher’s exact tests were used for the comparison of categorical variables. Wilcoxon rank-sum tests or Kruskal–Wallis *H* tests were used for the comparison of metadata and taxa relative abundance. Associations between mask wearing frequency or duration and microbiome taxonomic abundance data were analyzed with Spearman’s non-parametric correlation tests. The Benjamini–Hochberg procedure [false discovery rate (FDR)] was used to correct for multiple testing of taxon and metadata differences, with significance defined as FDR < 0.05.

## Results

### Demographics characteristics

A total of 82 prolonged mask wearers (mean age: 19.05 ± 0.68 years) and 172 mask-free controls (mean age: 18.89 ± 0.90 years) were enrolled. Average concentration of PM2.5 and PM10 in Shanghai in the past 6 months before sampling did not show significant difference in the two groups (**Data Sheet 4**). Moreover, there were no significant differences in age or sex between the masked and non-masked groups (**Table 1**). Every participant in 2023 self-reported as a prolonged mask user in the past 3 years. The average weekly duration of wearing masks for each masked participant was 16.06 ± 10.89 h in the previous 6 months; 46.34% (38/82) of these mask users wore masks for at least 16 h per week. With the relaxation of COVID-19 response by the Chinese government, a COVID-19 infection peak occurred in January 2023, just before our sampling time. The masked group was composed of 58.54% (48/82) COVID-19 convalescents and 41.46% (34/82) uninfected controls. Average interval between first COVID-19 infection time and sampling time was 44.50 ± 12.34 days; 97.56% (80/82) of participants in the masked group received at least two doses of COVID-19 inactive vaccine (**Data Sheet 2**).

**Table 1:** Demographics characteristics of participants.

	Non-masked	Masked	Masked				Weekly duration of wearing masks		
			*P*	Control	Recovery	*P*	Fewer than 16 hours / week	At least 16 hours / week	*P*
*n*	171	82		34	48		44	38	
Age	18.89±0.90	19.05±0.68	ns	19.15±0.70	18.98±0.67	ns	19.00±0.65	19.11±0.73	ns
Male	61 (35.67%)	28 (34.15%)		17 (50%)	11 (22.92%)		16 (36.36%)	12 (31.58%)	
Female	110 (64.33%)	54 (65.85%)	ns	17 (50%)	37 (77.08%)	*	28 (63.63%)	26 (68.42%)	ns

“ns” means P>0.05; “*” means P<0.05.

### Prolonged mask wearing changed nasal microbiota profile significantly

**Figure 1:**
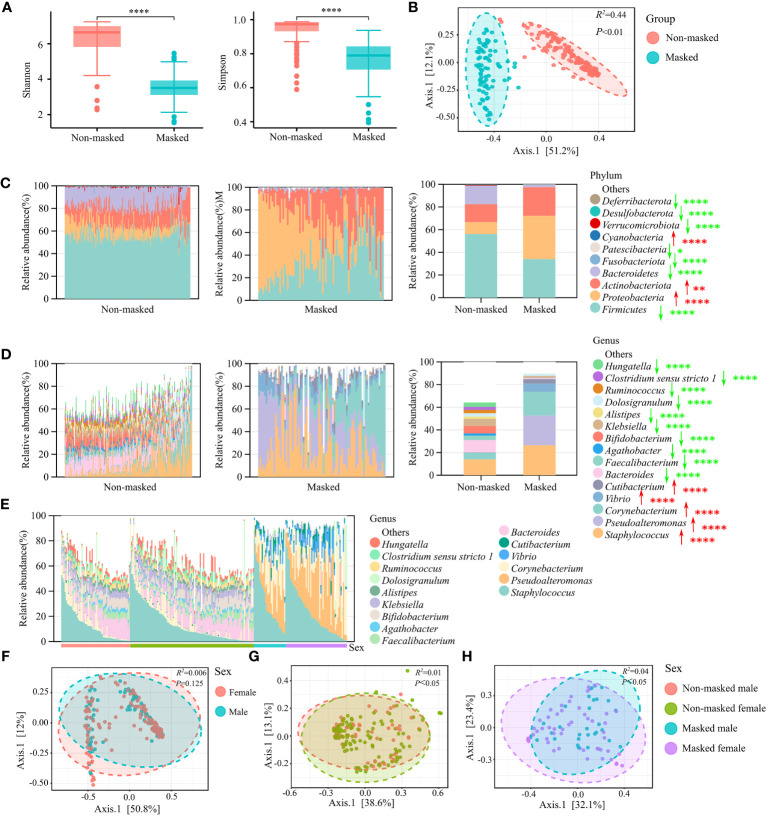
Nasal microbial characterization of prolonged mask wearers. **(A)** α-diversity (Shannon and Simpson indices) analysis of extensively masked group and mask-free group. “****” means *p* < 0.0001. **(B)** β-diversity (PCoA of Bray–Curtis distance) analysis of extensively masked group and mask-free group. **(C)** Relative abundance in phyla level. The left two panels show relative abundance in single individuals and the rightmost panel shows the average relative abundance of two groups. Red arrows signify significant increase in prolonged mask wearing group, and green arrows represent significant decrease. “*”, “**”, and “****” mean *p* < 0.05, *p* < 0.01, and *p* < 0.0001, respectively. **(D)** Relative abundance of the top 15 genera. The left two panels show relative abundance in single individuals and the rightmost panel shows the average relative abundance of two groups. Red arrows signify significant increase in prolonged mask wearing group, and green arrows represent significant decrease. “****” means *p* < 0.0001. **(E)** Relative abundance of top 15 genera in mask-free male, mask-free female, extensively masked male, and extensively masked female individuals. **(F)** β-diversity (PCoA of Bray–Curtis distance) analysis of male and female individuals. **(G)** β-diversity (PCoA of Bray–Curtis distance) analysis of male and female individuals in the mask-free group. **(H)** β-diversity (PCoA of Bray–Curtis distance) analysis of male and female individuals in the masked group.

To verify whether extended mask wearing changes the nasal microbial characteristics of the general population, we determined the nasal microbial profile of 82 prolonged mask wearers and 172 mask-free controls by 16S rRNA sequencing. The α-diversity was significantly lower in the masked than in the non-masked group (Shannon or Simpson diversity index, *p* < 0.0001) (**Figure 1A**), and there was a significant difference in β-diversity (PCoA of Bray–Curtis distance, *R* ^2 = ^0.44, *p* < 0.01) (**Figure 1B**).

Analysis of relative abundance at both phylum and genus level revealed significant changes in the abundance of almost all major taxonomic groups, indicating remarkable changes in the microbiome composition after long-term use of face masks (**Figures 1C, D**). The top four predominant phyla—*Proteobacteria*, *Actinobacteriota, Firmicutes*, and *Bacteroidetes*—composed over 99% of nasal microbiota in both groups. We observed significantly increased abundance of the *Proteobacteria* and *Actinobacteriota* phylum (FDR < 0.01), as well as decreased abundance of the *Firmicutes* and *Bacteroidetes* phylum (FDR < 0.0001) in the prolonged mask wearing group (**Figure 1C**). At the genus level, there were a total of 108 differential genera. Compared to the mask-free group, the abundance of some major genera including *Staphylococcus*, *Pseudoalteromonas*, *Corynebacterium*, *Vibrio*, and *Cutibacterium* was significantly increased (FDR < 0.0001) in the nares of prolonged mask wearers. Interestingly, the abundance of most genera such as *Bacteroides*, *Faecalibacterium*, *Agathobacter*, *Bifidobacterium*, and *Klebsiella* was significantly decreased (FDR < 0.0001) in the prolonged mask wearing group (**Figure 1D**). Furthermore, nasal microbiomes did not differ significantly by sex in the two groups (**Figures 1E, F**). Sex makes a contribution to the variation of nasal microbiota in both the non-masked and the masked population (**Figures 1G, H**). However, we did not observe significant differences of nasal microbiota between male and female individuals in the two sex-matched groups (**Figure 1F**).

**Figure 2:**
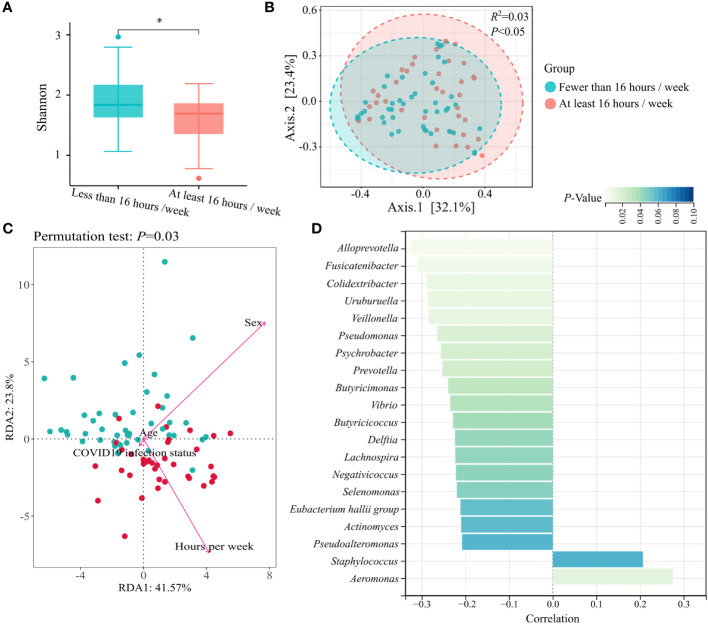
Nasal microbial characterization of prolonged mask wearers with different duration. **(A)** α-diversity (Shannon index) analysis grouped by mask wearing duration. “*” means *p* < 0.05. **(B)** β-diversity (PCoA of Bray–Curtis distance) analysis grouped by mask wearing duration. **(C)** Redundancy analysis ordination for the duration of wearing masks per week and other host factors in the masked group. All factors are represented by arrows. The length of the lines represents the degree of correlation between the factor and microbiota profile. **(D)** Top 20 genera mostly correlated with mask wearing duration. Genera with statistical differences are displayed in cyan (*p* < 0.05).

The microbiome composition was further compared by surveying the mask wearing status of the previous 6 months. We observed that duration of wearing masks influenced the diversity of nasal microbiota. Compared to wearing masks for fewer than 16 h per week, wearing masks for at least 16 h per week showed decreased α- diversity (Shannon diversity index, *p* < 0.05) (**Figure 2A**), as well as significant difference in β-diversity (PCoA of Bray–Curtis distance, *R* ^2 = ^0.03, *p* < 0.05) (**Figure 2B**). Redundancy analysis was used to illustrate the relationships between duration of wearing masks per week and other host factors, including age, sex, and COVID-19 infection status. The first two axes accounted for 65.37 of the variability. Sex (*R* ^2 = ^0.56, *p* < 0.001) and duration of wearing masks per week (*R* ^2 = ^0.44, *p* < 0.001) predominantly contributed to the variations of nasal microbiota profiles of the masked population (**Figure 2C**). After adjusting for sex covariate, the abundance of a total of 15 genera was negatively correlated with the weekly duration of mask wearing including *Alloprevotella*, *Fusicatenibacter*, and *Colidextribacter*, while *Aeromonas* was positively correlated with the duration of mask wearing (*p* < 0.05) (**Figure 2D**).

### The COVID-19 infection history did not affect the composition of nasal microbiota significantly

**Figure 3:**
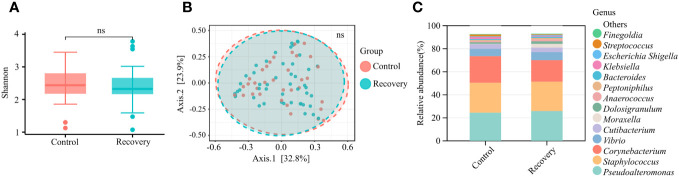
Nasal microbial characterization of COVID-19 convalescents. **(A)** α-diversity (Shannon index) analysis of COVID-19 convalescents and uninfected controls. “ns” means *p* > 0.05. **(B)** β-diversity (PCoA of Bray–Curtis distance) analysis of COVID-19 convalescents and uninfected controls. “ns” means *p* > 0.05. **(C)** Relative abundance of the top 15 genera.

With the purpose of verifying whether COVID-19 infection history affected the nasal microbiomes, the taxonomic abundance and composition of nasal microbiomes were further compared in uninfected and recovered young adults. There was no significant difference between microbial α-diversity or β-diversity (**Figures 3A, B**), and the microbial communities were similar in the two groups (**Figure 3C**). The results suggested COVID-19 convalescents displaying few changes in the nasal microbiota compared to uninfected individuals, indicating that COVID-19 infection history did not affect the composition of nasal microbiota significantly. After sex correction, no significant changes in nasal microbiota were observed either.

### The abundance of *Staphylococcus aureus* and *Corynebacterium accolens* increased in the nasal cavity of mask wearers

**Figure 4:**
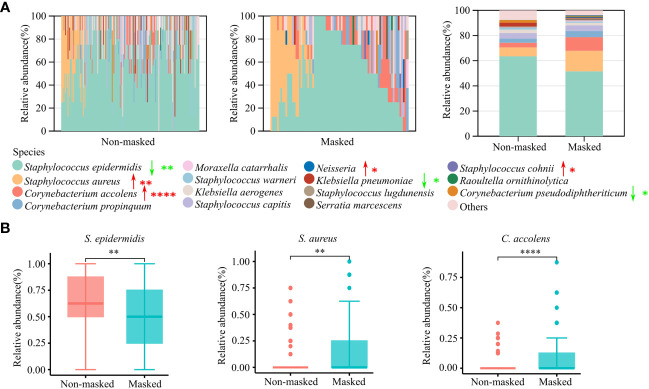
Culture-based microbiota analysis of prolonged mask wearers and mask-free controls. **(A)** Relative abundance of the top 15 cultured bacterial species. The left two panels show relative abundance in single individuals and the rightmost panel shows the average relative abundance of two groups. Red arrows signify a significant increase in prolonged mask wearing group, and green arrows represent significant decrease. “*”, “**”, and “****” mean *p* < 0.05, *p* < 0.01, and *p* < 0.0001, respectively. **(B)** Relative abundance of *S. epidermidis*, *S. aureus*, and *C*. *accolens*. “**” and “****” mean *p* < 0.01 and *p* < 0.0001, respectively.

By far, we observed significant differences in the composition of nasal microbiota by prolonged mask wearers. To further investigate how nasal microbiota was altered by wearing masks at the species level, we carried out culture-based analysis. A total of 43 bacterial species were successfully cultured and identified (**Data Sheet 2**). *S. epidermidis*, *S. aureus*, *Corynebacterium accolens*, *Corynebacterium propinquum*, and *S. capitis* were the major culturable species in the nasal cavity. Among them, the nasal colonization rate of *S. aureus* increased from 18.71% (32/171) to 31.71% (26/82) (*p* < 0.05), and *C. accolens* increased from 20.47% (35/171) to 42.68% (35/82) (*p* < 0.001) after extendedly wearing masks (**Data Sheet 3**), which were also confirmed by analysis of the relative abundance of all culturable bacteria (**Figures 4A, B**). We also observed the decrease of *S. epidermidis* and *Corynebacterium pseudodiphtheriticum* in the nares of the mask wearing population (*p* < 0.05) (**Figures 4A, B**; **Data Sheet 3**). Moreover, six species of *Streptococcus* genera (*S. oralis*, *S. infantarius*, *S. mitis*, *S. pneumoniae*, *S. pyogenes*, and *S. salivarius*) were isolated in the prolonged mask wearing group, and none in the non-masked group (**Data Sheet 3**). Taken together, culture-based analysis supported the alteration of nasal microbiota we observed via 16S rRNA sequencing.

### The abundant KEGG pathways were significantly affected by extended mask wearing

**Figure 5:**
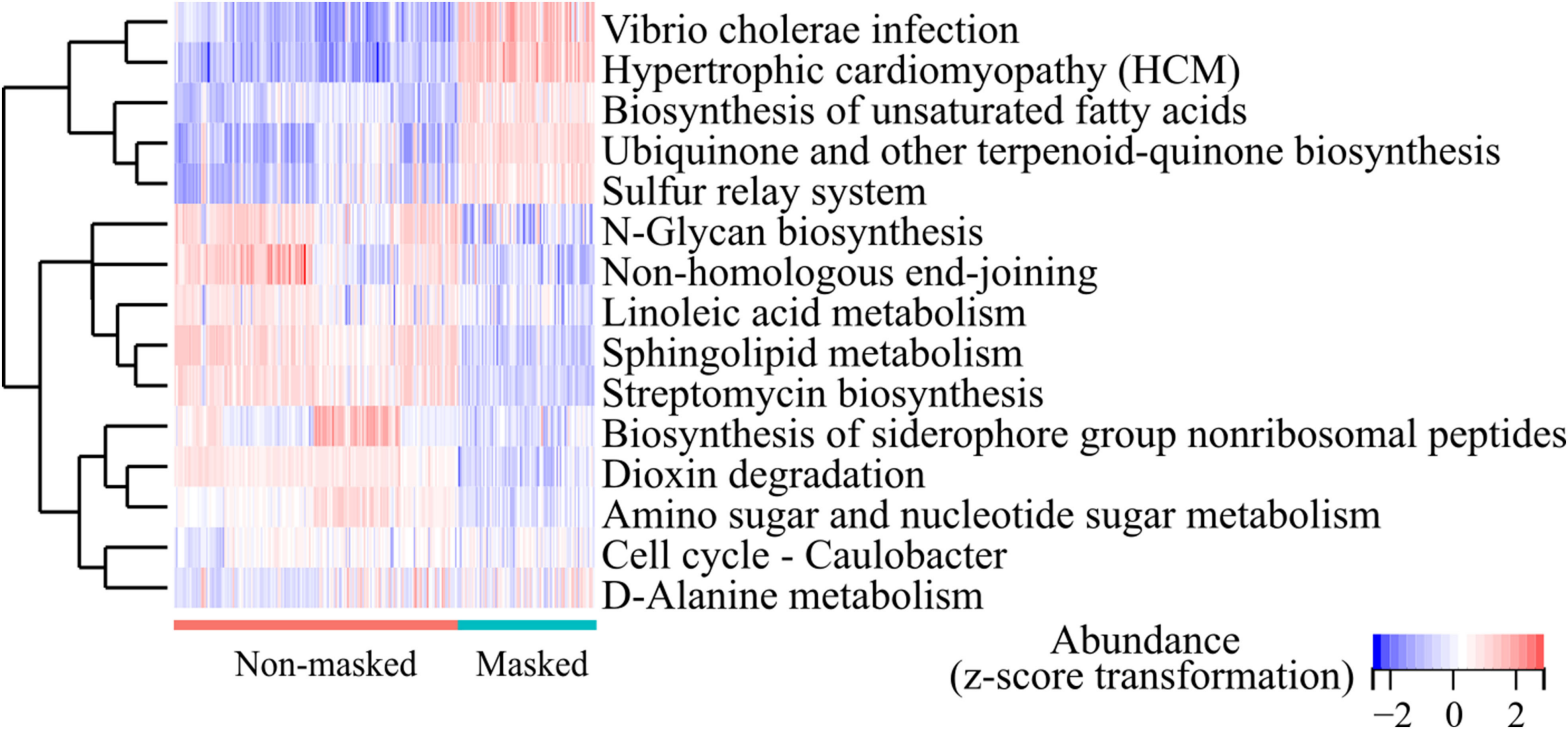
Top 15 differentially abundant KEGG pathways (level 3) between the extensively masked group and the mask-free group.

The metabolic process was associated with the composition of nasal microbiota (16). To access the potential functional changes in the microbiota after prolonged mask wearing, we employed PICRUSt2 for functional predictions. A total of 393 MetaCyc pathways and 157 KEGG pathways (level 3) were identified in all the samples. Prolonged mask wearing has extremely changed metabolization of nasal microbiomes; 65.61% (103/157) of KEGG pathways changed in abundance significantly (FDR < 0.0001), involving biosynthesis and metabolism of secondary metabolites, signal transduction, membrane transportation, and infectious diseases. The heatmap showed the 15 most differentially abundant KEGG pathways, displaying increased abundance of biosynthesis of unsaturated fatty acids and the D-Alanine metabolism pathway, as well as decreased abundance of the sphingolipid metabolism and linoleic acid metabolism pathway in the prolonged mask wearing group (**Figure 5**).

## Discussion

Nasal microbiota has crucial implications for human health. During the COVID-19 pandemic, face mask wearing was regarded as a necessary public health measure worldwide. This new lifestyle affects the perinasal microenvironment. It is essential to find out whether extended mask wearing changes the nasal microbial characteristics of the general population and the potential influence in medical fields. In this study, we observed a significant alteration of human nasal microbiota after long-term use of masks via the 16S rRNA sequencing method. Furthermore, culture-based analysis further revealed an increase of *S. aureus* and *C. accolens*, with a decrease of *S. epidermidis* in the nasal cavity compared to those in the mask-free periods.

The abundance of bacteria at the phylum level was affected significantly after wearing masks for a long time (**Figure 1**). The nasal microbiota plays a crucial role in the host immune responses, involved in the episode of diseases. Recently, it has been reported that increased *Proteobacteria* accompanied by decreased *Bacteroidetes* at the phylum level in nasal microbiota were involved in the pathogenesis of allergic diseases in China (17). Consistent changes were also observed in the nasal microbiota of prolonged mask wearers in our study, suggesting that long-term use of masks may bring potential risk of such disease. We further investigated the impact of mask usage duration on nasal microbiota in the past 6 months. Though sex could partially explain variations of nasal microbiota profiles of the masked population because of the hormone-shaped host environment (18), we still observed that duration of wearing masks influenced the diversity of nasal microbiota, further supporting the above finding that extensively wearing masks altered the composition of nasal microbiota (**Figure 2**).

The balance between commensal and pathogenic bacteria Is crucial for maintaining a healthy environment. Increasing studies have shown that commensal bacteria such as *S. epidermidis*, *C. pseudodiphtheriticum*, and *C. accolens* in the human nasal cavity could suppress opportunistic pathogen colonization including *S. aureus* and *S. pneumoniae* (5, 19, 20). Despite the potential antimicrobial activity, *C. accolens* could also cause opportunistic infections (21). The increased colonization of *C. accolens*, *S. aureus*, and *Streptococcus* in the nasal cavity after prolonged mask wearing, as observed in our study (**Figure 4** and **Data Sheet 3**), may pose a potential risk for various infections including soft-tissue infection, pneumonia, and bacteremia (21–23). Although masks could reduce the spread of aerosols, masks can also serve as a surface for the adherence of opportunistic pathogens originated from the oral cavity, nasal cavity, and skin (7). The warm and humid environment caused by mask wearing may contribute to the bacterial growth (7). The decreased abundance of some beneficial species such as *S. epidermidis* may also promote the proliferation of opportunistic pathogens (5). Therefore, proper mask hygiene is essential to prevent the growth and spread of opportunistic bacteria.

We observed great alterations in metabolization after long-term use of masks (**Figure 5**). Fatty acid metabolic pathway was significantly changed after wearing masks. Shifts in the composition of microbiota have the potential to impact the metabolism significantly (16). It is reported that *C. accolens* could release free fatty acids from human nostrils (20) and *S. aureus* could utilize host fatty acids to construct its membrane (24). The long-term impacts of metabolization changes of nasal microbiota are worth studying.

Our study observed that COVID-19 infection history did not affect the composition of nasal microbiota significantly (**Figure 3**). COVID-19 convalescents enrolled in our study were all asymptomatic and did not display symptoms of pneumonia. Whether COVID-19 infection would alter nasal microbiota composition is controversial (25, 26). Numerous cross-sectional or prospective studies have estimated the correlation of nasal microbiota with COVID-19 infection, severity, and prognosis by comparing characteristics of nasal microbiota between Severe Acute Respiratory Syndrome Corona 2 (SARS-CoV-2)-positive subjects and uninfected controls (27). However, the impacts of COVID-19 on nasal microbiota were not yet fully understood. It was reported that mild-to-moderate COVID-19 can lead to alterations of the upper respiratory tract microbiota that persist for several weeks after the initial infection (26), while other researchers observed that SARS-CoV-2 did not have a strong effect on the nasopharyngeal microbial composition (25). The COVID-19 infection on the nasal microbiome is a long-term event; it is necessary to monitor the microbiota dynamically.

There are some limitations in our study. We failed to recruit a group of mask-free control at the same time point because almost all people wear masks during the COVID-19 pandemic (28). The young people enrolled in 2023 have claimed to be long-term mask wearers since 2020, and the mask-free peers from the same school enrolled in 2018 were set as historical controls. Several longitudinal studies report that nasal microbiota remains relatively stable over time (29–31), suggesting that the great shifts of nasal bacterial community in our study were mainly caused by long-term use of masks instead of time fluctuations. In addition, self-reported mask wearing status could lead to potential misclassification bias. All recruitments in our study were college freshmen without occupational demands for wearing masks. However, people who occasionally wear a mask could not be fully excluded in the mask-free group. Last but not least, our research was conducted among young adults, excluding children and the elderly. It is worth evaluating whether long-term use of masks affected the maturation of children’s nasal microbiota in the future, and further prospective studies are needed to investigate the long-term effects of the observed microbial changes.

In summary, our data indicated significant and remarkable changes in the nasal microbiome composition after long-term use of face masks, with increasing colonization rates of *S. aureus* and *C. accolens* in nasal cavity, changing metabolic pathways extensively. To better understand the relationship between face mask and the nasal microbiome, the potential impact of these alterations on host physiology and pathophysiology needs to be further investigated.

## Data availability statement

The supplementary materials presented in this study can be found in the article/**Supplementary Material**. Raw microbiome sequencing data have been deposited in NCBI’s Sequencing Read Archive (SRA) database under Bioproject numbers PRJNA508588 and PRJNA978391, respectively.

## Ethics statement

The studies involving humans were approved by the ethics committee of Renji Hospital, School of Medicine, Shanghai Jiaotong University, Shanghai, China. The studies were conducted in accordance with the local legislation and institutional requirements. Written informed consent for participation in this study was provided by the participants’ legal guardians/next of kin.

## Author contributions

GX: Formal analysis, Writing – original draft. KX: Investigation. YJ: Investigation. LH: Software. ZS: Data curation. ML: Conceptualization, Writing – review & editing. QL: Conceptualization, Writing – review & editing.
