# Supplementary material for: Prolonged mask wearing changed nasal microbial characterization of young adults during the COVID-19 pandemic in Shanghai, China
Source: Front Immunol. 2023 Oct 16;14:1266941. doi: 10.3389/fimmu.2023.1266941 (PMC10614009; doi:10.3389/fimmu.2023.1266941)
Supplement: Supplementary file 4 [file DataSheet_4.docx]

Figure S1. Everage concentration of PM2.5 and PM10 in the 6 months before sampling.


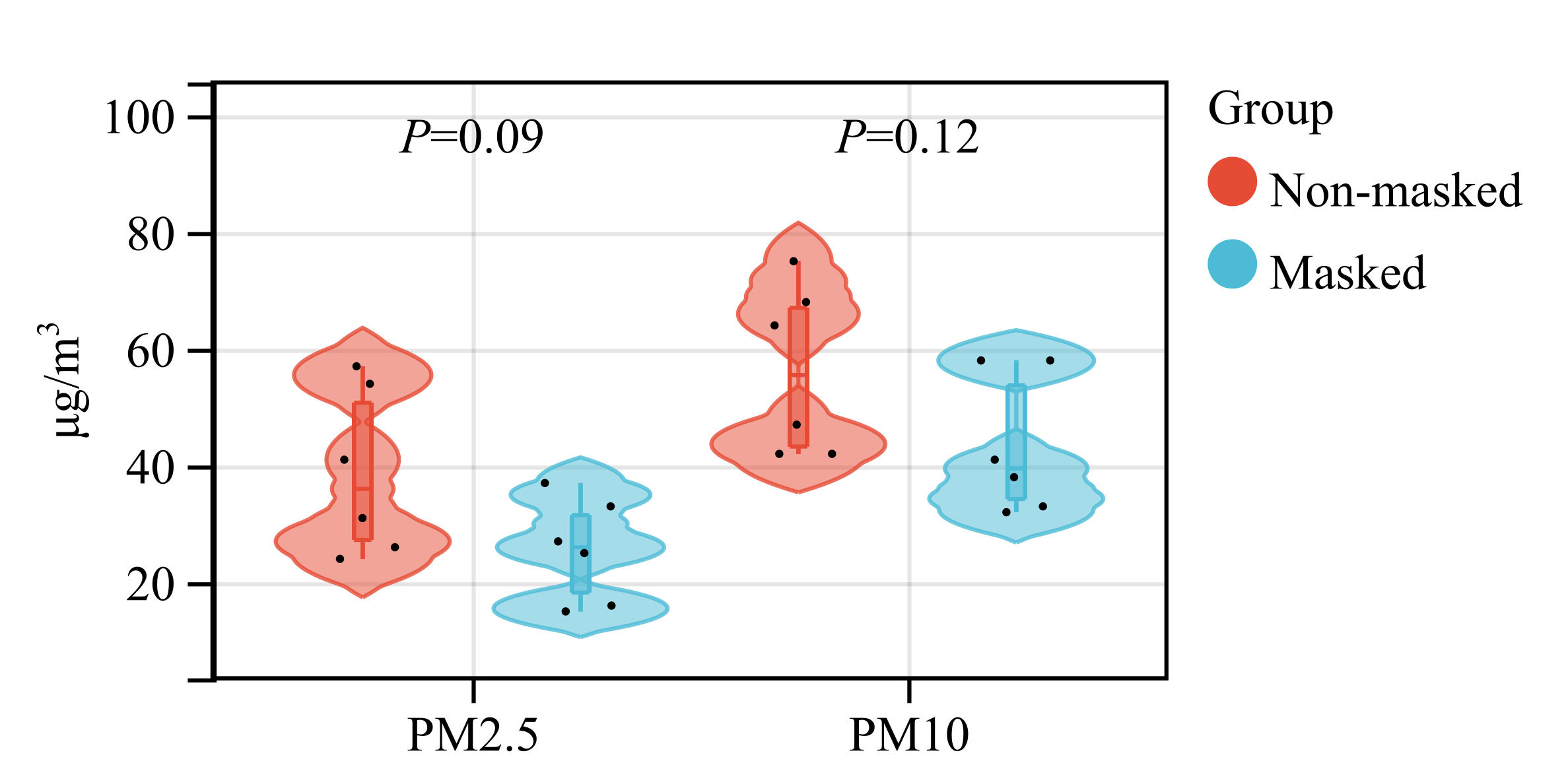


The data was sourced from government statistics (https://www.aqistudy.cn/).
